# Association between red blood cell distribution width-to-platelet ratio and post-discharge readmission rate in patients with heart failure: A retrospective cohort study

**DOI:** 10.1016/j.heliyon.2024.e26549

**Published:** 2024-02-16

**Authors:** Shan Lin, Xueyan Mao, Wanmei He, Qingyuan Zhan

**Affiliations:** a Department of Respiratory and Critical Care Medicine, Affiliated Hospital of North Sichuan Medical College, Nanchong, Sichuan, China; b Department of Medical Intensive Care Unit, The First Affiliated Hospital of Sun Yat-sen University, Guangzhou, Guangdong, 510080, China; c Department of Pulmonary and Critical Care Medicine, Center of Respiratory Medicine, National Center for Respiratory Medicine, China-Japan Friendship Hospital, Beijing, China

**Keywords:** Heart failure, Prognosis, Readmission, Inflammatory marker

## Abstract

**Background:**

To date, no studies have investigated the association between red blood cell distribution width (RDW)-to-platelet ratio (RPR) and readmission rates among patients with heart failure (HF). As such, the present study aimed to examine the relationship between RPR and readmission rates in patients with HF.

**Methods:**

Data for this study were obtained from the Fourth People's Hospital (Zigong, Sichuan Province, China). Patients were diagnosed with HF in accordance with European Society of Cardiology criteria. The primary outcome was the 28-day readmission rate. Various logistic regression models were constructed to explore the association between RPR and the 28-day readmission rate.

**Results:**

The study comprised 1978 patients with HF, with a 28-day readmission rate of 6.98%. RPR emerged as an independent risk factor for 28-day readmission, evidenced by consistent results across the various regression-adjusted models. The covariate-adjusted propensity score model demonstrated that every 0.1 increase in RPR was associated with an 8.2% increase in 28-day readmission rate (odds ratio [OR] 1.082 [95% confidence interval (CI) 1.012–1.158]; *P* = 0.0212). Similarly, each 0.1 change in RPR was associated with a 9.8% (OR 1.098 [95% CI 1.014–1.188]) and 7.3% (OR 1.073 [95% CI 0.991–1.161]) increase in 3- and 6-month readmission rates, respectively. However, RPR was not statistically associated with the 6-month readmission rate. Curve fit plots illustrated a nonlinear positive correlation between RPR and 28-day, and 3- and 6-month readmissions. Moreover, the effects of RPR on 28-day, and 3- and 6-month readmission rates remained robust across subgroup variables in stratified analysis. Finally, the effect sizes of pooled multiply imputed data were consistent with the original data, suggesting robust results.

**Conclusion:**

RPR was an independent risk factor for 28-day readmission among patients with HF and also demonstrated modest predictive value for readmissions at 3 and 6 months, despite being non-significant for the 6-month readmission rate. Early identification of patients with HF with elevated RPR would facilitate management and may confer favorable effects on prognosis.

## 1 Introduction

The growing aging population and advances in modern therapies that prolong the lives of patients with cardiovascular disease have led to an increasing prevalence of heart failure (HF). Despite technological improvements in treatment, mortality rates remain remarkably high among patients with HF, underscoring the urgent need to identify susceptible populations earlier that may benefit from preventive interventions [1]. A study of the Chinese Hypertension Survey found that approximately 1.3% (13.7 million) of adults ≥35 years of age had HF [2]. Moreover, Chinese HF guidelines indicate a 6-fold increase in HF prevalence over the past decade, with mortality rates approaching 50% in patients with severe HF [3]. The China-HF study reported a 4.1% in-hospital mortality rate for hospitalized patients with HF [4]. In the United States, total deaths attributable to HF increased from 275,000 in 2009 to 310,000 in 2014 [5]. The rise in hospitalizations has coincided with improved post-diagnosis survival in HF, resulting in proportional growth of HF hospitalizations and readmissions, although readmission rates continue to challenge contemporary HF care. One study reported that >20% of patients were readmitted within 30 days, and up to 50% by 6 months, despite current advances and developments in HF management [6].

Biomarker-guided management has introduced a new dimension to prediction, diagnosis, and treatment selection. Based on the recommendation of natriuretic peptides (brain natriuretic peptide [BNP] and N-terminal-pro BNP), many other biomarkers have been thoroughly investigated to reflect the different pathophysiological processes (e.g., fibrosis, inflammation, myocardial injury, and remodeling) in HF, and some of these biomarkers have been subsequently recommended to aid in the diagnosis and prediction of HF [[7], [8], [9], [10]]. Among the markers targeting the inflammatory response, C-reactive protein, interleukin (IL)-1, IL-6, tumor necrosis factor-alpha (TNF-α), Fas (APO-1), procalcitonin, and other inflammatory markers have also been shown in studies to be associated with prognosis in patients with HF [[11], [12], [13]]. However, none of these cytokines have been able to provide satisfactory conclusions to drive the transition to daily clinical use in HF. Nevertheless, predicting readmission rates for HF remains an important ongoing topic, closely tied to patient prognosis.

Routine blood testing is one of several convenient, standard laboratory investigations performed in hospitals. Red blood cell distribution width (RDW) indicates red blood cell volume heterogeneity and aids morphological classification of anemia. Current studies have identified RDW as a marker of inflammation that predicts adverse outcomes across various diseases, including HF, sepsis, cancer, and acute kidney injury [[14], [15], [16], [17]]. Beyond their vital role in coagulation, platelets also mediate key inflammatory and immune responses. Through endothelial adhesion, platelets facilitate neutrophil chemotaxis, infiltration and proinflammatory factor production during acute inflammation [18]. The RDW-to-platelet ratio (RPR) is a novel, easily obtained indicator. Several studies have examined associations between RPR and prognosis across diseases [[19], [20], [21]]. However, to our knowledge, none have investigated the relationship between RPR and readmission rates among patients with HF. The present study, therefore, aimed to examine the association between RPR and readmission rates in patients with HF after hospital discharge.

## 2 Materials and methods

### 2.1 Study design and setting

A retrospective cohort study using patient data from patients hospitalized for HF was conducted. The data were collected at the Fourth People's Hospital of Zigong (Sichuan Province, China) between December 2016 and June 2019, and are maintained in an ongoing manner by the Massachusetts Institute of Technology (Cambridge, MA, USA; https://physionet.org/content/heart-failure-zigong/1.3/) [22]. Access to the database was granted by the institutional review boards of Beth Israel Deaconess Medical Center (Boston, MA, USA) and the Massachusetts Institute of Technology Affiliates (Record ID: 49780033). This study was also approved by the ethics committee of Zigong Fourth People's Hospital (Approval number: 2020–010). Data were finalized on June 8, 2020, and were completely anonymized. The study complied with the Declaration of Helsinki and, because the data were anonymized, requirements for informed consent were waived.

### 2.2 Subjects

All patient information was obtained from the Zigong HF database (version 1.3), and the diagnosis of HF was made in accordance with the criteria from the European Society of Cardiology (i.e., “ESC”) [23], as follows: presence of symptoms and/or signs of HF, with typical symptoms such as breathlessness, orthopnea, paroxysmal nocturnal dyspnea, reduced exercise tolerance, fatigue, increased time to recover after exercise, and ankle swelling. Typical signs include elevated jugular venous pressure, hepatojugular reflux, third heart sound (gallop rhythm), and laterally displaced apical impulse; elevated levels of natriuretic peptides (BNP >35 pg/mL and/or NT-proBNP >125 pg/mL); and objective evidence of other cardiac functional and structural alterations underlying HF. In case of uncertainty, a stress test or invasively measured elevated left ventricular filling pressure may have been needed to confirm the diagnosis.

### 2.3 Variables

Baseline clinical parameters were measured on the day of admission, including age, sex, laboratory investigation results, and examinations. New York Heart Association (NYHA) cardiac function classification was assessed based on clinical presentation [24], and left ventricular ejection fraction (LVEF) was assessed using echocardiography. In the raw data, age (in years) was categorized into four groups: >21 to ≤ 49; >49 to ≤ 69; >69 to ≤ 89; and >89 to ≤ 110.

### 2.4 Covariate filtering methods

In the initially adjusted model (model I), two conventional confounders―age and sex―were adjusted for. The screening process for covariates in the final model was as follows. First, covariate screening was performed to exclude variables with a variance inflation factor (VIF) > 5. Next, a covariate was included as a potential confounder if it resulted in a change >10% in the RPR on 28-day, 3-month, and 6-month readmission rate estimates, or was significantly associated with 28-day, 3-month, and 6-month readmission rates. LVEF was not included in the final adjusted model because it did not have a significant effect on either the exposure or outcome values. Multiple imputation (MI) based on 5 replications was used, and a chained equation approach method in the R MI procedure was used to account for missing data [25]. The final effect values were pooled using the 5 data sets generated by MI.

### 2.5 Outcomes

The primary endpoint was 28-day readmission rate, while the secondary endpoints were the 3- and 6-month readmission rates.

### 2.6 Statistical analysis

Continuous variables are expressed as mean ± standard deviation (SD) or median (interquartile range [IQR]), whereas categorical variables are expressed as number and percentage. Clinical characteristics between the two groups were compared using chi-squared tests for categorical variables, and Student's *t*-test or Wilcoxon rank-sum test for continuous variables for 28-day readmission. To examine the relationship between RPR and 28-day readmission rate, three multivariate logistic regression models were constructed: model I was adjusted for patient demographic factors (age and sex); model II added adjustments for degree of HF (i.e., NYHA classification), Charlson Comorbidity Index (CCI) scores, admission HF indicators (BNP, high-sensitivity troponin [Hs-troponin]), and medication use (e.g., angiotensin-converting enzyme inhibitor [ACEI]/angiotensin-receptor blocker [ARB], β-blocker, diuretic) to model I; and model III adjusted propensity scores for the aforementioned covariates. For the secondary outcomes (3- and 6-month readmission rates), only models I and II were used. Generalized additive models were also used to depict the relationship between RPR and the primary and secondary outcomes. Finally, whether the effects of RPR remained consistent across subgroups of variables for the primary and secondary outcomes were explored using interaction tests and stratified analyses. All data were analyzed using EmpowerStats (www.empowerstats.com) and R (R Foundation for Statistical Computing, Vienna, Austria <http://www.R-project.org>). Differences with P < 0.05 were considered to be statistically significant.

## 3 Results

### 3.1 Characteristics of the study population

**Fig. 1:**
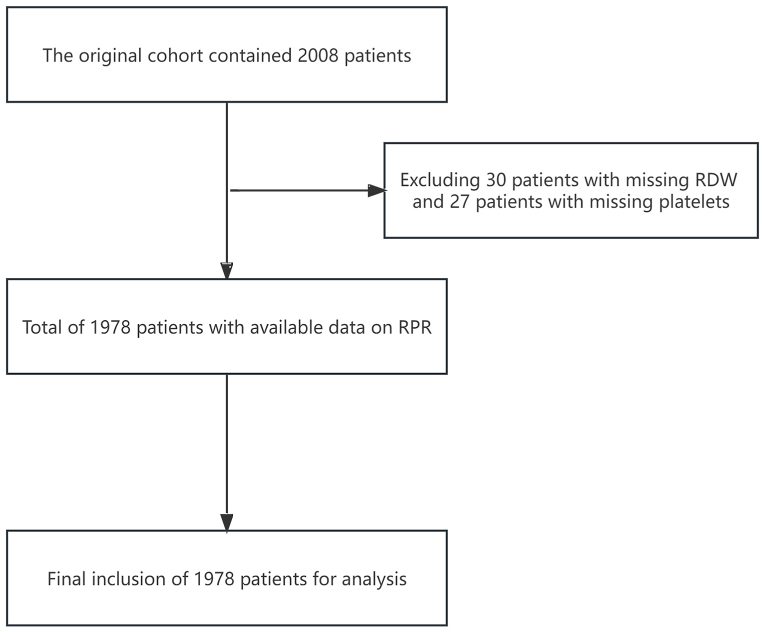
Flow chart **Abbreviations:** RDW, red blood cell distribution width; RPR, RDW to platelet ratio.

The study included 1978 patients with HF divided into two groups based on 28-day readmission status (Fig. 1). The groups did not significantly differ in age category or sex (Table 1). Among the total study population, the majority of patients were 69–89 years of age, with a predominance of females. Most patients were NYHA class III, and 98.53% were treated with diuretics followed by cardiac stimulants. Of the admission indices, only Hs-troponin exhibited a significant between-group difference. Median Hs-troponin level was substantially higher in readmitted versus non-readmitted patients. No statistically significant differences were observed between the groups for LVEF, BNP, RDW, platelets, or RPR. Similarly, CCI scores did not differ significantly. However, patients with NYHA class IV exhibited markedly higher 28-day readmission rates. Finally, regarding medications, non-readmitted patients had notably higher anticoagulant and ACEI/ARB usage compared with readmitted patients. Additional details are summarized in Table 1.

**Table 1:** Characteristics of participants.

28-day re-admission	All (N = 1978)	No (N = 1840)	Yes (N = 138)	*P*-value
Age, n (%)				0.804
>21, ≤49	70 (3.54%)	66 (3.59%)	4 (2.90%)	
>49, ≤69	463 (23.41%)	434 (23.59%)	29 (21.01%)	
>69, ≤89	1345 (68.00%)	1246 (67.72%)	99 (71.74%)	
>89, ≤110	100 (5.06%)	94 (5.11%)	6 (4.35%)	
Sex, n (%)				0.104
Male	830 (41.96%)	763 (41.47%)	67 (48.55%)	
Female	1148 (58.04%)	1077 (58.53%)	71 (51.45%)	
LVEF (%) [mean ± SD]	50.45 ± 13.54	50.43 ± 13.58	50.70 ± 13.05	0.832
BNP (pg/ml) [IQR]	759.65 (308.37–1749.18)	743.90 (307.36–1735.45)	1014.12 (310.66–1948.15)	0.106
Hs-Troponin (pg/ml) [IQR]	0.05 (0.02–0.12)	0.05 (0.02–0.12)	0.07 (0.03–0.21)	<0.001
RDW (%) [IQR]	14.40 (13.60–15.60)	14.40 (13.60–15.60)	14.60 (13.70–15.67)	0.412
Platelet (10^9^/L) [IQR]	135.00 (101.00–177.00)	135.00 (101.00–177.00)	133.00 (93.00–180.50)	0.633
RPR [IQR]	0.11 (0.08–0.15)	0.11 (0.08–0.15)	0.11 (0.08–0.16)	0.672
NYHA classification, n (%)				0.004
II	341 (17.24%)	327 (17.77%)	14 (10.14%)	
III	1027 (51.92%)	961 (52.23%)	66 (47.83%)	
IV	610 (30.84%)	552 (30.00%)	58 (42.03%)	
CCI [IQR]	2.00 (1.00–2.00)	2.00 (1.00–2.00)	2.00 (1.00–3.00)	0.149
Medication, n (%)				
Antiplatelet drug use	1181 (59.74%)	1105 (60.09%)	76 (55.07%)	0.247
Anticoagulation use	475 (24.03%)	452 (24.58%)	23 (16.67%)	0.036
ACEI/ARB use	759 (38.39%)	721 (39.21%)	38 (27.54%)	0.007
β-blocker use	754 (38.14%)	711 (38.66%)	43 (31.16%)	0.080
Cardiotonic use	1738 (87.91%)	1611 (87.60%)	127 (92.03%)	0.124
Diuretic use	1948 (98.53%)	1813 (98.59%)	135 (97.83%)	0.474
Statin use	810 (40.97%)	756 (41.11%)	54 (39.13%)	0.648

**Abbreviations:** BNP, brain natriuretic peptide; Hs-Troponin, high sensitivity troponin; RDW, red blood cell distribution width; RPR, red blood cell distribution width to platelet ratio; NYHA, New York Heart Association; CCI, charlson comorbidity index; LVEF, left ventricular ejection fractions; ACEI/ARB, angiotensin converting enzyme inhibitor/angiotensin receptor blocker.

### 3.2 Associations between RPR and primary and secondary outcomes

**Fig. 2:**
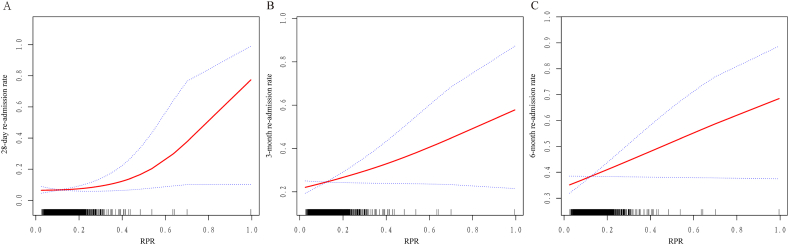
Associations of RPR with primary and secondary outcomes. **Note:** adjusted by age, sex, NYHA classification, LVEF, hs-Troponin, BNP, antiplatelet drug use, anticoagulation use, ACEI/ARB use, β-blocker use, cardiotonic use, diuretic use, statin use. **Abbreviations:** BNP, brain natriuretic peptide; Hs-Troponin, high sensitivity troponin; NYHA, New York Heart Association; CCI, charlson comorbidity index; LVEF, left ventricular ejection fractions; ACEI/ARB, angiotensin converting enzyme inhibitor/angiotensin receptor blocker.

Regarding the primary outcome, RPR was found to be an independent risk factor for 28-day readmission (Table 2). In regression-adjusted models, elevated RPR exhibited a significant positive association with 28-day readmission, with consistently robust results across all models. The propensity score-adjusted model demonstrated that each 0.1 increase in RPR was associated with an 8.2% increase in 28-day readmission (odds ratio [OR] 1.082 [95% confidence interval (CI) 1.012–1.158]; *P* = 0.0212) (Table 2). For secondary outcomes, each 0.1 change in RPR was associated with a 9.8% (OR 1.098 [95% CI 1.014–1.188]) and 7.3% (OR 1.073 [95% CI 0.991–1.161]) increase in 3- and 6-month readmission rates, respectively (Table 3). However, RPR was not statistically associated with the 6-month readmission rate. In addition, curve-fitting plots illustrating the relationship between RPR and 28-day, 3-month, and 6-month readmissions demonstrated that an increase in RPR was strongly associated with an increase in readmission rates at all time points (Fig. 2 A–C).

**Table 2:** Associations between RPR and primary outcome.

28-day re-admission rate	OR (95% CI)	OR (95% CI) (Per 0.1 increase)	*P*-value
Crude	2.24 (1.14–4.39)	1.084 (1.013–1.159)	0.0189
Model I	2.13 (1.08–4.20)	1.079 (1.008–1.154)	0.0283
Model II	2.10 (1.05–4.23)	1.077 (1.005–1.155)	0.0367
Model III	2.21 (1.13–4.33)	1.082 (1.012–1.158)	0.0212

**Note:** Model I was adjusted by age, sex. Model II was adjusted by age, sex, CCI score, NYHA classification, hs-Troponin, BNP, antiplatelet drug use, anticoagulation use, ACEI/ARB use, β-blocker use, cardiotonic use, diuretic use, statin use.

Model III was adjusted by propensity score calculated by age, sex, CCI score, NYHA classification, hs-Troponin, BNP, antiplatelet drug use, anticoagulation use, ACEI/ARB use, β-blocker use, cardiotonic use, diuretic use, statin use.

**Abbreviations:** BNP, brain natriuretic peptide; Hs-Troponin, high sensitivity troponin; NYHA, New York Heart Association; CCI, charlson comorbidity index; ACEI/ARB, angiotensin converting enzyme inhibitor/angiotensin receptor blocker; OR, odds ratio; CI, confidence interval.

**Table 3:** Associations between RPR and secondary outcomes.

Outcome	Crude (OR, 95% CI, *P*-value)	Model I (OR, 95% CI, *P*-value)	Model II (OR, 95% CI, *P*-value)
3-month re-admission rate	2.83 (1.24–6.47) 0.0135	2.71 (1.23–6.00) 0.0137	2.54 (1.15–5.58) 0.0205
3-month re-admission rate (Per 0.1 increase)	1.110 (1.022–1.205) 0.0135	1.105 (1.021–1.196) 0.0137	1.098 (1.014–1.188) 0.0205
6-month re-admission rate	2.37 (1.04–5.39) 0.0399	2.25 (1.01–5.01) 0.0465	2.02 (0.92–4.45) 0.0809
6-month re-admission rate (Per 0.1 increase)	1.090 (1.004–1.184) 0.0399	1.085 (1.001–1.175) 0.0465	1.073 (0.991–1.161) 0.0809

**Note:** Model I was adjusted by age, sex. Model II was adjusted by age, sex, CCI score, NYHA classification, hs-Troponin, BNP, antiplatelet drug use, anticoagulation use, ACEI/ARB use, β-blocker use, cardiotonic use, diuretic use, statin use.

**Abbreviations:** BNP, brain natriuretic peptide; Hs-Troponin, high sensitivity troponin; NYHA, New York Heart Association; CCI, charlson comorbidity index; ACEI/ARB, angiotensin converting enzyme inhibitor/angiotensin receptor blocker; OR, odds ratio; CI, confidence interval.

### 3.3 Effect size of RPR on primary and secondary outcomes in prespecified and exploratory subgroups in each subgroup

Stratified analysis and interaction tests revealed that the effect of RPR on 28-day readmission remained robust across subgroup variables (Table 4). Similarly, in stratified analyses of 3- and 6-month readmission rates, RPR exhibited a consistent influence on readmissions at both time points for each subgroup variable (Table 5).

**Table 4:** Effect size of RPR on 28-day re-admission rate in prespecified and exploratory subgroups in each subgroup.

28-day re-admission	Crude		Adjusted model		
RPR	OR (95% CI)	*P*-value	OR (95% CI)	*P*-value	*P* interaction
Age category					0.9521
>21, ≤69	2.22 (0.88–5.64)	0.0920	1.62 (0.58–4.47)	0.3547	
>69, ≤110	2.30 (0.86–6.15)	0.0962	2.11 (0.78–5.72)	0.1425	
Sex					0.4103
Male	2.19 (1.10–4.35)	0.0250	2.85 (1.34–6.06)	0.0067	
Female	0.75 (0.01–40.09)	0.8865	0.20 (0.00–15.55)	0.4690	
NYHA classification					0.4970
II	1.32 (0.20–8.59)	0.7707	1.75 (0.24–12.69)	0.5783	
III	2.83 (1.13–7.09)	0.0260	2.85 (1.08–7.53)	0.0349	
IV	3.43 (0.49–24.28)	0.2163	2.23 (0.28–17.93)	0.4496	
BNP (pg/ml)					0.9201
<100	1.10 (0.00–57085.18)	0.9865	0.13 (0.00-a)	0.8271	
≥100	2.27 (1.16–4.47)	0.0171	2.17 (1.06–4.42)	0.0336	
Hs-Troponin (pg/ml)					0.1495
<0.06	6.17 (0.77–49.26)	0.0861	7.49 (0.95–58.73)	0.0555	
≥0.06	1.61 (0.70–3.73)	0.2651	1.52 (0.62–3.70)	0.3562	

**Note:** Adjusted model adjust for: age, sex, CCI score, NYHA classification, hs-Troponin, BNP, antiplatelet drug use, anticoagulation use, ACEI/ARB use, β-blocker use, cardiotonic use, diuretic use, statin use.

**Abbreviations:** BNP, brain natriuretic peptide; Hs-Troponin, high sensitivity troponin; NYHA, New York Heart Association; CCI, charlson comorbidity index; ACEI/ARB, angiotensin converting enzyme inhibitor/angiotensin receptor blocker; OR, odds ratio; CI, confidence interval.

a The model failed because of the small sample size.

**Table 5:** Effect size of RPR on secondary outcomes in prespecified and exploratory subgroups in each subgroup.

Subgroup variables	Adjusted model			Adjusted model		
RPR	3-month re-admission OR (95% CI)	*P*-value	*P* interaction	6-month re-admission OR (95% CI)	*P*-value	*P* interaction
Age category			0.0762			0.1251
>21, ≤69	2.43 (0.63–9.39)	0.1978		2.24 (0.47–10.72)	0.3131	
>69, ≤110	2.42 (0.87–6.72)	0.0900		1.86 (0.71–4.86)	0.2065	
Sex			0.3905			0.8100
Male	3.56 (1.27–9.99)	0.0159		2.24 (0.90–5.56)	0.0835	
Female	0.78 (0.08–7.96)	0.8315		1.26 (0.16–9.69)	0.8216	
NYHA classification			0.4157			0.1329
II	1.54 (0.48–4.94)	0.4630		0.94 (0.25–3.56)	0.9290	
III	6.36 (0.98–41.23)	0.0525		6.75 (0.95–47.66)	0.0557	
IV	2.59 (0.43–15.41)	0.2969		3.37 (0.50–22.84)	0.2127	
BNP (pg/ml)			0.8496			0.5481
<100	14.22 (0.00–177981.92)	0.5813		0.03 (0.00–103.51)	0.4071	
≥100	2.43 (1.10–5.36)	0.0273		2.09 (0.92–4.73)	0.0786	
Hs-Troponin (pg/ml)			1.0000			1.0000
<0.06	6.59 (0.81–53.60)	0.0780		25.54 (2.64–247.33)	0.0052	
≥0.06	1.86 (0.79–4.38)	0.1528		1.22 (0.56–2.65)	0.6155	

**Note:** Adjusted model adjust for: age, sex, CCI score, NYHA classification, hs-Troponin, BNP, antiplatelet drug use, anticoagulation use, ACEI/ARB use, β-blocker use, cardiotonic use, diuretic use, statin use.

**Abbreviations:** BNP, brain natriuretic peptide; Hs-Troponin, high sensitivity troponin; NYHA, New York Heart Association; CCI, charlson comorbidity index; ACEI/ARB, angiotensin converting enzyme inhibitor/angiotensin receptor blocker; OR, odds ratio; CI, confidence interval.

### 3.4 Sensitivity analysis

The effect of RPR on 28-day, 3-month, and 6-month readmission rates was explored using MI of 5 datasets and integrating the effect values generated from the 5 imputed datasets. This analysis suggested that after MI, the effect of RPR on 28-day, 3-month, and 6-month readmission rates remained consistent with the original data, indicating robust results (Table 6).

**Table 6:** Association between RPR and readmission rates at 28-day, 3-month, and 6-month by MI data.

Outcome	28-day re-admission rate	3-month re-admission rate	6-month re-admission rate
MI data 1 (OR, 95% CI, *P*-value)	2.06 (1.03–4.14) 0.0420	2.47 (1.14–5.35) 0.0221	1.96 (0.91–4.25) 0.0871
MI data 2 (OR, 95% CI, *P*-value)	2.06 (1.02–4.13) 0.0430	2.48 (1.14–5.37) 0.0213	1.96 (0.91–4.22) 0.0867
MI data 3 (OR, 95% CI, *P*-value)	2.06 (1.02–4.13) 0.0427	2.48 (1.14–5.37) 0.0216	1.98 (0.91–4.29) 0.0852
MI data 4 (OR, 95% CI, *P*-value)	2.05 (1.02–4.12) 0.0434	2.46 (1.14–5.33) 0.0222	1.96 (0.90–4.24) 0.0881
MI data 5 (OR, 95% CI, *P*-value)	2.04 (1.01–4.09) 0.0456	2.46 (1.14–5.32) 0.0224	1.96 (0.90–4.24) 0.0881
Pooled OR from MI data	2.05 (1.02–4.13) 0.0434	2.47 (1.14–5.35) 0.0218	1.96 (0.91–4.25) 0.0869

**Note:** Models adjust for: age, sex, CCI score, NYHA classification, hs-Troponin, BNP, antiplatelet drug use, anticoagulation use, ACEI/ARB use, β-blocker use, cardiotonic use, diuretic use, statin use.

**Abbreviations:** BNP, brain natriuretic peptide; Hs-Troponin, high sensitivity troponin; NYHA, New York Heart Association; CCI, charlson comorbidity index; ACEI/ARB, angiotensin converting enzyme inhibitor/angiotensin receptor blocker; OR, odds ratio; CI, confidence interval.

MI: multiple imputation.

## 4 Discussion

The present study explored the effect of RPR on readmission rates among patients with HF. We found that elevated RPR was significantly associated with high readmission rates, which may inform the care of patients with HF. Early screening of patients prone to readmission may have favorable implications for prognosis.

RDW refers to a simple parameter obtained from routine blood tests that reflects the degree of variation in red blood cell size. Traditionally, RDW has been studied in relation to anemia etiology. However, mounting evidence indicates that elevated RDW is closely linked to poor prognosis across various diseases, including sepsis, coronary artery disease, and HF [15,[26], [27], [28]]. It has been established that HF involves activation of proinflammatory cytokines. Elevated IL-1β, TNF-α, and IL-6 can inhibit erythropoietin (EPO)-induced erythrocyte maturation, contributing to increased RDW [29]. While the relationship between anemia and HF outcomes also involves inflammatory stress and insufficient EPO production, any of these mechanisms could underlie anemia and RDW elevation in HF [30,31]. Patients with HF are highly prone to anemia, with a previous study reporting that 37.2% of 153,180 patients with chronic HF had anemia. During the 6-month follow-up, 46.8% of anemic patients died versus 29.5% of non-anemic patients and, after adjusting for confounders, anemia was identified as an independent risk factor in chronic HF [32,33]. The potential links between inflammatory response and elevated RDW in HF were hypothesized based on previous studies. However, we did not analyze correlations between inflammatory markers, such as ILs and RDW, to elucidate the underlying mechanisms, due to unavailable data regarding these inflammatory mediators.

Platelets represent an intersection between immune response and coagulation function. A recent study reported that platelet indices were linked to impaired cardiac function and adverse clinical outcomes, with low platelet count related to reduced LVEF and poorer patient prognosis [34]. Platelets are recognized mediators of inflammation [35]. Platelet count, platelet-to-monocyte ratio, and platelet-to-lymphocyte ratio (PLR) have emerged as novel, readily obtainable markers of inflammation with predictive value for both inflammatory status and disease prognosis. Several studies have demonstrated the diagnostic utility of PLR in HF, with higher PLR strongly associated with poor clinical outcomes among patients with HF [[36], [37], [38], [39]].

As an inflammatory marker, RPR has also been extensively studied in hepatic and pancreatic diseases, among others [40,41]. In our previous study of RPR in sepsis, we observed an association between high RPR and poor prognosis [42]. The link between RPR and HF warrants further investigation. Mechanistically, inflammatory response plays a pivotal role in the pathogenesis of HF [43]. Leukocytes and their subtypes are salient inflammatory markers in cardiovascular diseases [44]. Inflammatory stimuli prompt leukocytes to release many proinflammatory cytokines, such as TNF-α, IL-6, C-reactive protein, and specific protein hydrolases [45]. These inflammatory mediators inflict damage on the myocardium, resulting in decreased left ventricular function and eventual HF [43,46]. Previous studies have reported that elevated levels of proinflammatory cytokines can lead to myocardial remodeling and arrhythmias [47,48]. The increase in RDW can be attributed to activated inflammatory response in HF on the one hand, and increased inflammatory factors, including IL-1β, TNF-α, and IL-6 that suppress erythropoiesis thereby elevating RDW on the other, as well as insufficient EPO production in HF that causes anemia, also contributing to elevated RDW. Additionally, platelet abnormalities have been extensively characterized in HF. Platelet activation participates in both inflammatory and coagulation pathways, and cardiac chamber dilation, impaired contractility, localized wall motion abnormalities. Moreover, concomitant atrial fibrillation can instigate thromboembolism by promoting blood stagnation within the heart [49,50]. Elevated plasma coagulation marker levels in patients with reduced ejection fraction suggest that diminished cardiac output may promote a prothrombotic or hypercoagulable state [51,52]. Furthermore, platelets participate in inflammatory response(s) by inducing the release of specific cytokines, such as TNF-α and macrophage chemotactic protein-1 [53,54]. Therefore, altered bone marrow function induced by HF and inflammation, together with platelet consumption due to hypercoagulation or blood thrombosis, contributes to decreased platelet count [55]. In summary, the combination of increased RDW and reduced platelet count leads to abnormal RPR. To date, RPR has been scarcely studied in cardiac diseases. For the first time, we have probed the relationship between RPR and readmission rates in patients with HF. Overall, this study offers an important preliminary investigation of RPR in HF readmissions; however, ongoing research is imperative to validate and extend these findings, unravel the intricate biology linking RPR with HF outcomes, and explore the clinical utility of leveraging RPR to refine risk stratification and care for this high-risk patient population.

The present study had some limitations that should be noted. Primarily, we lacked data regarding the impact(s) of inflammation, anemia and EPO on RDW, precluding detailed analysis of the underlying mechanisms linking these factors with RDW. Although we used initial blood counts on admission to minimize treatment effects, the influences of long-term medication regimens on HF outcomes still require further elucidation. Additionally, as a single-center investigation, the external validity and generalizability of our findings to other settings may be limited. We were also unable to examine the effects of temporal RPR changes on outcomes due to absence of serial blood count measurements. Moving forward, further efforts are imperative to better characterize the biological associations between high RPR and adverse outcomes, delineate interrelationships between RPR and HF anemia/inflammation and, importantly, determine whether resolving anemia and inflammation improves RPR. In particular, elucidating the intriguing possibility of mitigating elevated RPR through anemia and inflammation correction could profoundly impact prognostication and management in HF.

## 5 Conclusion

RPR was an independent risk factor for 28-day readmission among patients with HF, and also demonstrated modest predictive value for readmissions at 3 and 6 months, despite being non-significant for the 6-month readmission rate. Early identification of patients with HF with elevated RPR would facilitate management and may confer favorable effects on prognosis.

## Data availability statement

The raw data itself is from a third-party dataset. Reproduction of their data is not permitted according to the Data Use Agreement of the database but access can be requested here: https://physionet.org/content/heart-failure-zigong/1.3/.

## Ethics statement

The access of the database has been approved by the institutional review boards of both Beth Israel Deaconess Medical Center and Massachusetts Institute of Technology Affiliates (Record ID: 49780033). Also, this study was approved by the ethics committee of Zigong Fourth People's Hospital (Approval Number: 2020–010).

## Funding

This work was supported by the Key Project of the Affiliated Hospital of North Sichuan Medical College (2023ZD008).

## CRediT authorship contribution statement

**Shan Lin:** Writing – review & editing, Writing – original draft, Funding acquisition, Formal analysis, Data curation, Conceptualization. **Xueyan Mao:** Writing – original draft, Formal analysis. **Wanmei He:** Visualization, Formal analysis. **Qingyuan Zhan:** Writing – original draft, Data curation.

## Declaration of competing interest

The authors declare that they have no known competing financial interests or personal relationships that could have appeared to influence the work reported in this paper.
